# The effects of ursodeoxycholic acid on albuminuria in people with type 1 diabetes and diabetic kidney disease: a proof-of-concept study

**DOI:** 10.3389/fendo.2026.1908356

**Published:** 2026-09-24

**Authors:** Nikolaos Fountoulakis, Panagiotis Pavlou, Luigi Gnudi, Giuseppe Maltese, Angeliki Panagiotou, Salma Ayis, Fowzia Ibrahim, Janaka Karalliedde

**Affiliations:** 1 School of Cardiovascular and Metabolic Medicine and Sciences, King’s College London, London, United Kingdom; 2 School of Life Course and Population Sciences, King’s College London, London, United Kingdom; 3 King’s College London, British Heart Foundation Centre of Excellence, School of Cardiovascular and Metabolic Medicine and Sciences, King’s College London, London, United Kingdom

**Keywords:** albuminuria, clinical trial, diabetic kidney disease, type 1 diabetes, ursodeoxycholic acid

## Abstract

**Introduction:**

In animal models of diabetic kidney disease (DKD), ursodeoxycholic acid (UDCA) reduces albuminuria and improves histological changes associated with DKD. The purpose of this study was to investigate the potential renoprotective role of UDCA in people with type 1 diabetes (T1D) and DKD.

**Methods:**

In this single-centre, double-blind, placebo-controlled crossover trial, participants with T1D and DKD with residual albuminuria (urine albumin to creatinine ratio, > 3 mg/mmol) on a maximum tolerated, stable dose of renin–angiotensin system (RAS) blockade with estimated glomerular filtration rate (eGFR) ≥ 30 ml/min were included. Participants were randomly allocated to UDCA (500 mg BID) or placebo for 24 weeks, with a 4-week washout period in between. The primary endpoint was the change in urine albumin excretion rate (UAER), measured from timed urine collections after 24 weeks of treatment with UDCA compared with placebo.

**Results:**

Thirty-one participants were included, of whom 21 were men; the median age (interquartile range [IQR]) was 54.2 (22.7–75.3) years. The baseline median (IQR) UAER was 26 (12.3–189.0) μg/min for UDCA and 32.1 (19.9–88.2) μg/min for placebo. The median (IQR) posttreatment UAER was 36.2 (14.9–174.0) μg/min for UDCA and 38.5 (17.6–194.0) μg/min for placebo, with no significant difference in UAER between treatments (*p*-value = 0.4).

**Conclusion:**

In this first-ever human study investigating the potential renoprotective effect of UDCA treatment for 24 weeks in people with T1D and DKD, we did not observe a significant reduction in UAER. Further studies are required to evaluate the role of UDCA in DKD.

**Clinical trial registration:**

https://www.clinicaltrialsregister.eu/ctr-search/trial/2015-003609-41, identifier 2015-003609-41.

## Highlights

- What was known
  - Residual albuminuria persists in many people with type 1 diabetes despite renin–angiotensin system blockade, leaving substantial unmet need for new treatments.
  - Ursodeoxycholic acid reduced albuminuria and improved the histological features of diabetic kidney disease in animal models.
  - Before this study, no human trial had evaluated ursodeoxycholic acid in people with diabetic kidney disease.

- This study adds
  - We investigated the effect of ursodeoxycholic acid on urinary albumin excretion rate, compared with placebo, in a single-centre, double-blind, placebo-controlled crossover trial involving adults with type 1 diabetes and residual albuminuria receiving RAS inhibition.
  - No significant benefits were seen with ursodeoxycholic acid treatment for the primary endpoint or secondary exploratory endpoints, including eGFR, systolic blood pressure, HbA1c, or aortic pulse wave velocity.

- Potential impact
  - These findings do not support the routine use of ursodeoxycholic acid to reduce albuminuria in people with type 1 diabetes and diabetic kidney disease receiving standard renin–angiotensin system blockade.
  - This study provides the first feasibility data in people with type 1 diabetes and diabetic kidney disease to inform future studies evaluating the role of ursodeoxycholic acid as a potential renoprotective treatment.

## Background

Diabetic kidney disease (DKD) is the leading cause of dialysis or renal transplantation amongst people with type 1 diabetes (T1D) (1, 2). One of the earliest manifestations of DKD is albuminuria (increased urinary albumin excretion), which indicates a high risk of progression towards end-stage kidney disease (ESKD) requiring dialysis or transplantation. Albuminuria is also an index of endothelial dysfunction that characterises DKD and a powerful independent predictor of cardiovascular disease (CVD) (1).

DKD develops in 30%–40% of people with type 1 diabetes, and 10%–15% will develop ESKD in their lifetime (1, 3). People with T1D and albuminuria (urine albumin-to-creatinine ratio, ≥ 3 mg/mmol; albumin excretion rate, ≥ 20 µg/min) are at an increased risk of progression to ESKD, with up to a 20-fold higher risktowards (4). Despite recent improvements in clinical care and treatment with renin–angiotensin system (RAS) inhibitors, between 35% and 52% of people with T1D and albuminuria progress to ESKD over 10–15 years of follow-up in European and US studies (5). Additionally, in the “Preventing Early Renal Loss in Diabetes (PERL)” study, the placebo group participants with albumin-creatinine-ratio (ACR) > 10 mg/mmol experienced a rate of reduction in estimated glomerular filtration rate (eGFR) > 3 ml/min/year despite RAS inhibition, indicative of significant kidney function loss and disease progression even at lower levels of urine albumin excretion rate (UAER) (6). More effective novel therapies are urgently needed to either prevent or slow down the progression to ESKD in this large number of people. Recent data support the use of finerenone as a treatment for DKD in addition to RAS inhibition, with albuminuria used as the study endpoint on the basis of its value as a bridging biomarker endpoint for kidney disease events (7).

Indeed, UAER is well established as a biomarker of DKD progression risk and a predictor of treatment response (8, 9). Treatments that reduce albuminuria result in a significant reduction in the progression of DKD towards ESKD (1). In people with DKD and macroalbuminuria, a 50% reduction in albuminuria in the first 24 weeks of treatment translates to a 45% reduction in the long-term risk of ESKD (3, 10, 11), and recent data suggest that in high-risk individuals, any degree of reduction in albuminuria would delay progression towards ESKD and may also reduce CVD (3, 11).

Ursodeoxycholic acid is a naturally occurring bile acid secreted in human bile and is currently used for the treatment of gallstones and liver disease associated with cholestasis. Laboratory work on rodent models of DKD demonstrated that ursodeoxycholic acid treatment reduces albuminuria and improves the histological changes of DKD by reducing endoplasmic reticulum (ER) stress and insulin resistance, novel pathways that drive renal damage (12, 13). These results were independent of changes in blood pressure, lipids, or glucose. In addition, ursodeoxycholic acid treatment for 4 weeks has been shown to improve endothelial function in people with stable chronic heart failure (14). It is worth noting that enhanced ER stress response and endothelial dysfunction have been implicated in the pathogenesis of DKD and, as such, both constitute potential therapeutic targets for ursodeoxycholic acid (15, 16). To date, there have been no studies evaluating the effects of ursodeoxycholic acid in people with DKD. Our objective was to evaluate whether ursodeoxycholic acid reduces albuminuria in people with T1D who had residual albuminuria despite established standard care with RAS inhibition.

## Methods

In this single-centre, double-blind, placebo-controlled crossover trial, participants with T1D and DKD with residual albuminuria (urine albumin-to-creatinine ratio, > 3 mg/mmol) on a maximum tolerated, stable dose of RAS blockade with eGFR ≥ 30 ml/min were included. Participants were randomly allocated to ursodeoxycholic acid (500 mg twice daily) or placebo for 24 weeks, with a 4-week washout period in between.

The primary endpoint was the change in albuminuria measured by timed UAER after 24 weeks of treatment with ursodeoxycholic acid compared with placebo. Secondary endpoints included blood pressure and eGFR, as well as other independent predictors and biomarkers of renal and CVD risk, such as glycated haemoglobin (HbA1c), total cholesterol, and triglyceride levels.

The inclusion criteria were people with T1D aged 20 to 75 years with residual albuminuria (defined as a urine albumin-to-creatinine ratio, > 3 mg/mmol) or proteinuria in the preceding 6 months whilst receiving a maximum tolerated, stable dose of RAS blockade, such as angiotensin-converting enzyme (ACE) inhibitor or angiotensin receptor blocker (ARB), an estimated GFR (by the MDRD equation) ≥ 30 ml/min, written informed consent to participate in the study before any study procedures, and ability to communicate and comply with all study procedures.

Exclusion criteria included a history of intolerance to ursodeoxycholic acid, active gastrointestinal disease (such as gallstones, inflammatory bowel disease, or primary sclerosing cholangitis), nondiabetic renal disease, absence of diabetic retinopathy, pregnancy, insufficient understanding of the trial, and lactation.

The primary endpoint was UAER after 24 weeks of treatment with ursodeoxycholic acid as compared with placebo. UAER data were log-transformed for analysis. The null hypothesis (H0) tested was H0: *μ*_ursodeoxycholic acid_ − *μ*_placebo_ = 0, where *μ* is the mean endpoint value for log-transformed UAER for ursodeoxycholic acid and placebo.

For the primary endpoint, UAER values were analysed using analysis of covariance (ANCOVA), with baseline UAER as a covariate, using a modified intention-to-treat (ITT) approach that included all randomised participants who received at least one dose of a study drug and had at least one baseline measurement of the primary outcome. To account for the crossover design, analyses estimated the effects of treatment and period. The least squares means, 95% confidence intervals (CI), and *p*-value for the intervention effect were estimated. The possibility of an interaction between treatment and period, which might indicate a carryover effect, was evaluated graphically to inform future trial design. A potential interaction between treatment and baseline UAER value was similarly evaluated. Missing data were handled by listwise deletion, and no imputation was performed.

Secondary endpoints were evaluated in the same framework, including mixed models to confirm whether the results observed with ANCOVA were consistent. Additional analyses evaluated potential baseline prognostic factors associated with UAER changes over time. The list of potential prognostic variables at baseline was determined before the final analysis plan.

No major changes to the trial plan were made, but of note, there was a suspension of study recruitment and visits due to the coronavirus disease 2019 (COVID-19) pandemic, with related changes made to the protocol to enable remote visits/assessments. The primary endpoint was not changed, nor were the methods for its assessment. There was no missing data for UAER. However, due to limitations in sample collection/visits, we could not collect all the required data for the secondary exploratory and exploratory endpoints. No imputation was performed for missing data for these secondary endpoints. The total amount of missing data was < 30%.

Treatment adherence was monitored through protocol-specified compliance assessments that included tablet counting at the end of each treatment period. Individual and overall drug accountability were conducted by the clinical trials pharmacy and independently verified during monitoring visits. Participants were instructed to return all unused study medication at the completion of each treatment period.

The analysis was undertaken on log-transformed data with an aim to detect a 30% reduction in the mean value, as previously observed (1, 4). This equates to a difference in log values of ln(550) − ln(385) = ln(550/385) = 0.36. Assuming an SD of 0.63 for the log UAER values, as reported previously (4), for 80% power and a 5% significance level, 26 participants were required. Factoring in a 15% dropout rate (observed by our group and others in trials of similar duration in people with DKD (1, 4)), we aimed to recruit 30 people in total.

The dose selected for ursodeoxycholic acid was 500 mg twice daily (BID). This was based on the dose (500 mg BID) used in a study involving people with stable chronic heart failure (stage II/III) and another trial of 11 people with ischaemic heart disease (ursodeoxycholic acid dose, 13–19 mg/kg/day; ~ 1,000 mg/day), both of which showed improved endothelial function and good tolerability with ursodeoxycholic acid (14, 17).

The 4-week washout period was based on data from studies suggesting a half-life of less than 6 days and the fact that other washout studies using ursodeoxycholic acid for cardiac endpoints used similar doses to those used in our study (18).

The trial was conducted according to the protocol and in compliance with the principles of the Declaration of Helsinki (1996), as amended, the principles of Good Clinical Practice (GCP), and the Medicines for Human Use (Clinical Trials) Regulations 2004, the Research Governance Framework for Health and Social Care, the Data Protection Act 1998, and other regulatory requirements. The trial protocol and substantial amendments were reviewed by the United Kingdom (UK) Medicines and Healthcare products Regulatory Agency (MHRA). All participants gave informed consent prior to undergoing any study procedures or the screening visit. The first participant was recruited on 31 March 2017 and the last one on 20 February 2020. The COVID-19 disruption occurred between 16 March 2020 and 29 March 2021, during which some study visits were converted to virtual visits; however, all primary endpoint data were collected from the per-protocol population.

## Results

A total of 45 individuals underwent screening assessment, of whom 14 were excluded either due to noneligibility (*n* = 10) or withdrawal of consent prior to starting the intervention (*n* = 4). Subsequently, 31 participants were randomised to two groups, either ursodeoxycholic acid followed by placebo (*n* = 16) or placebo followed by ursodeoxycholic acid (*n* = 15), and were eligible for primary endpoint analysis. The intervention was discontinued in seven participants over the course of the study (**Figure 1**). The final analysis included 31 participants, all of whom were eligible for the modified ITT analyses. Of the cohort, 24 participants fully completed the trial by attending all visits according to the protocol (**Figure 1**).

**Figure 1:**
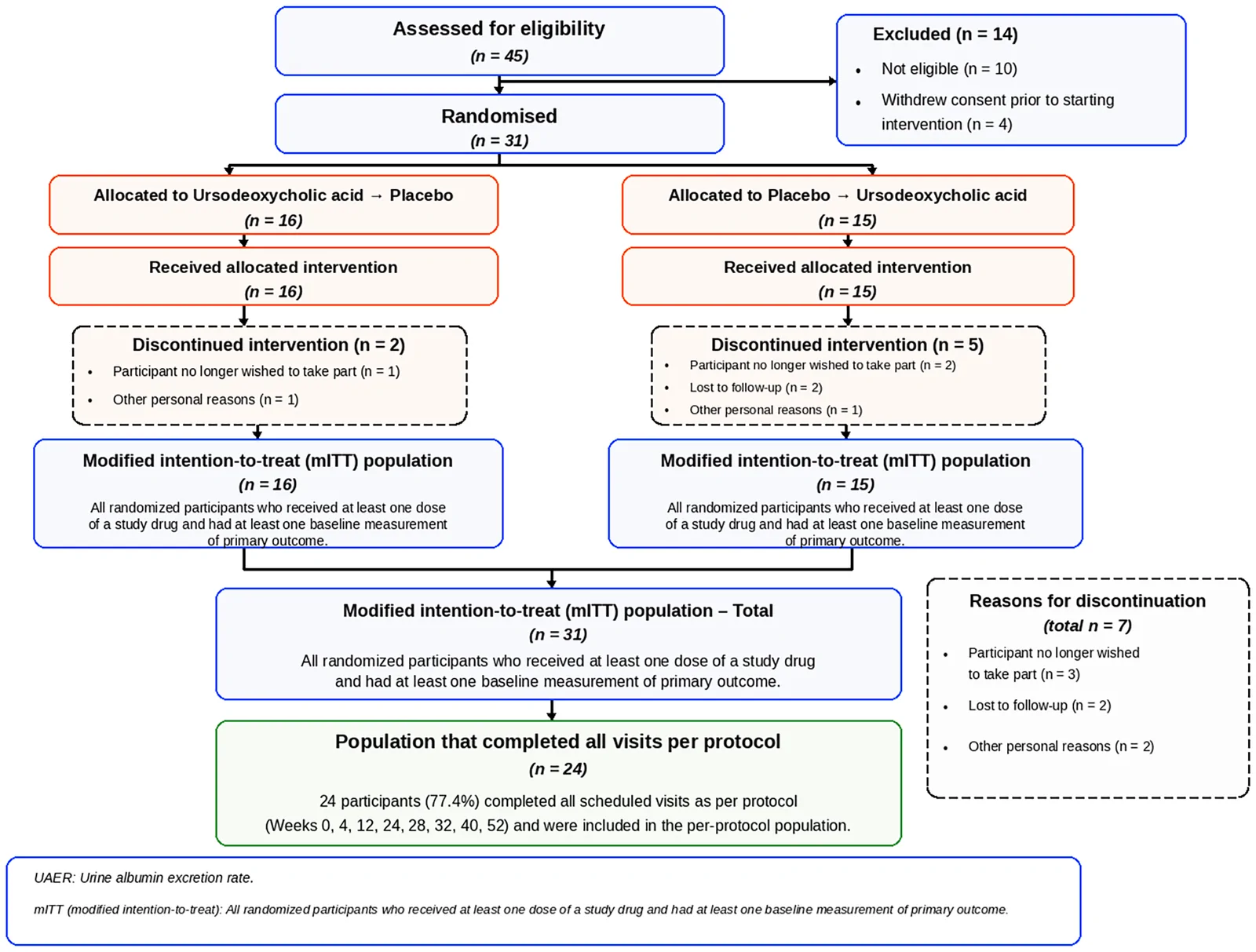
Consort diagram outlining the study methodology.

Regarding participant demographics and baseline characteristics, the median age (range) was 54.2 (22.7–75.3) years, and the median diabetes duration (range) was 31.3 (11.8–59.5) years. For the study as a whole, 67.7% of participants were men, and 32.3% were women; 87.1% were White, 6.5% Asian, 3.2% Black, and 3.2% mixed. The baseline features of participants at each phase/stage of the crossover study are shown in **Table 1**.

**Table 1:** Clinical characteristics of participants before each treatment period according to treatment sequence.

Characteristic	UDCA → placebo week 0 (*n* = 16)	Placebo → UDCA week 0 (*n* = 15)	*p*-value	UDCA → placebo week 28 (*n* = 15)	Placebo → UDCA week 28 (*n* = 12)	*p*-value
UAER (μg/min; median, IQR)	23.0 (12.0–31.0)	30.0 (19.0–62.5)	0.357	35.0 (22.8–322.0)	55.5 (17.8–237.5)	1.000
eGFR (ml/min/1.73 m^2^; mean ± SD)	81.1 ± 27.3	96.3 ± 63.4	0.404	79.4 ± 27.1	84.0 ± 32.1	0.740
Systolic BP (mmHg; mean ± SD)	131.4 ± 18.7	132.1 ± 17.1	0.910	132.9 ± 19.7	118.3 ± 20.3	0.117
Diastolic BP (mmHg; mean ± SD)	73.0 ± 9.9	76.1 ± 9.3	0.378	74.2 ± 13.8	80.3 ± 13.7	0.303
HbA1c (mol/mol; mean ± SD)	66.0 ± 10.7	71.6 ± 15.3	0.266	65.5 ± 14.5	70.7 ± 16.4	0.467
Aortic PWV (m/s; median, IQR)	8.2 (6.7–9.8)	8.0 (6.5–9.8)	1.000	8.9 (6.8–12.7)	7.4 (6.5–11.1)	0.750
Total cholesterol (mol/L; mean ± SD)	4.36 ± 0.80	4.05 ± 1.00	0.364	4.72 ± 1.55	4.69 ± 1.40	0.966
Triglycerides (mmol/L; median, IQR)	0.90 (0.90–1.48)	1.10 (0.65–1.85)	0.878	0.85 (0.67–1.23)	1.00 (0.57–1.52)	0.938

Data are presented as mean ± SD for approximately normally distributed variables and median (IQR) for skewed variables. Welch’s independent-samples *t*-test for normally distributed variables and the Mann–Whitney *U* test for skewed variables were used for comparisons. All tests were two-sided, with *p* < 0.05 considered statistically significant.

*BP*, blood pressure; *eGFR*, estimated glomerular filtration rate; *HbA1c*, glycated haemoglobin; *IQR*, interquartile range; *PWV*, pulse wave velocity; *SD*, standard deviation; *UAER*, urine albumin excretion rate; *UDCA*, ursodeoxycholic acid.

All participants were on a stable dose of RAS blockade for > 4 weeks prior to entry, and this remained consistent during the trial. The medications used were ACE inhibitors in 63.3% of participants and ARBs in 36.7%, with stable doses maintained throughout the study. Other medications, including statins, which were used in 62.5% of participants, and other antihypertensives, used in 37.5% (25.0% calcium-channel blockers, 12.5% beta-blockers, 12.5% loop diuretics, 3.1% thiazide diuretics, and 3.1% centrally acting antihypertensives), remained stable during the trial. No nsMRA or MRA was used, and no participants were on SGLT-2 inhibitors or GLP-1 RA. Other baseline features and clinical characteristics of the study cohort are shown in **Supplementary Table 1**.

The primary endpoint was the change in UAER after 24 weeks of treatment with ursodeoxycholic acid compared with 24 weeks on placebo in the modified ITT population (*n* = 31). ANCOVA was conducted to assess the effect of treatment on end-of-study UAER whilst adjusting for baseline UAER. The model showed a small reduction in UAER; however, the treatment effect was not statistically significant after baseline adjustment (estimate = − 0.3, SE = 0.3; 95% CI, − 0.8 to 0.3; *p* = 0.4), as shown in **Supplementary Table 2**. In contrast, baseline UAER was a strong predictor of end-of-study UAER (*F*(1, 39) = 106.40; *p* < 0.001). These findings indicate that whilst baseline UAER strongly influenced the outcome, there was no significant treatment effect on final UAER. **Figure 2** shows the trajectory of UAER with ursodeoxycholic acid versus placebo, by treatment sequence, over the study period.

**Figure 2:**
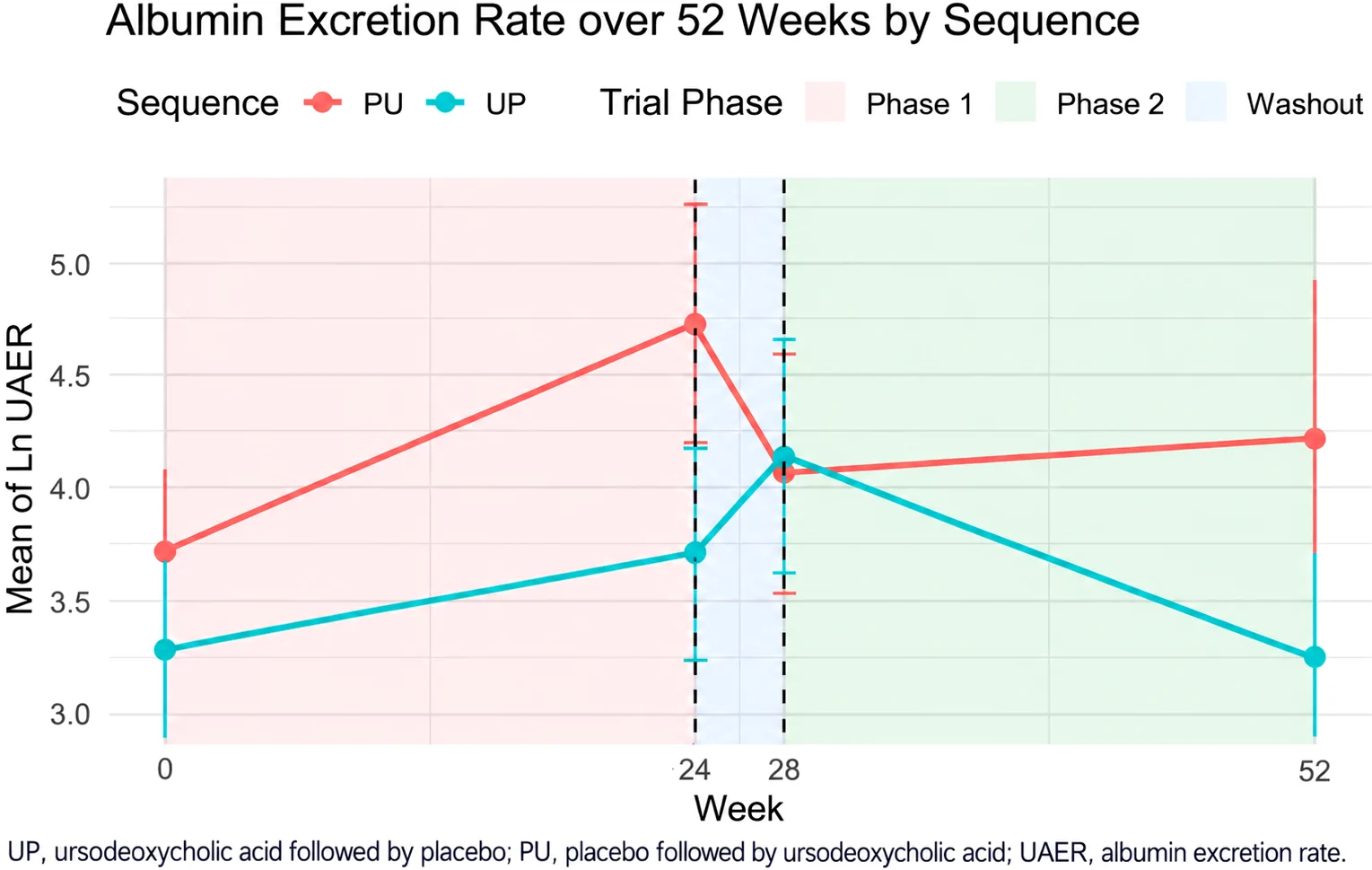
Trajectory of UAER by sequence from baseline to week 52. PU, Placebo-Ursodeoxycholic acid; UP, Ursodeoxycholic acid-Placebo; UAER, Urine Albumin Excretion Rate.

To evaluate the assumptions of the crossover design, as part of the supporting secondary analyses, a mixed-effects model was applied to examine treatment, period, and sequence effects on the primary endpoint. The treatment effect was not statistically significant (estimate = − 0.1, SE = 0.1; *p* = 0.5). The period effect was small and nonsignificant (estimate = 0.1, SE = 0.1; *p* = 0.3), indicating no systematic influence of study period on the outcome. Likewise, the sequence effect was not significant (estimate = − 0.5, SE = 0.6; *p* = 0.4), providing no evidence of carryover effects. Collectively, these findings support the validity of the crossover design and confirm that neither period nor carryover effects biased the analysis of the primary endpoint (**Supplementary Table 3**).

For secondary exploratory endpoint analyses at week 24, the same analytical approach as that used for the primary endpoint was applied, using ANCOVA. For each secondary endpoint, the adjusted model included the change from baseline for the endpoint as the dependent variable, with treatment group and the corresponding baseline value as explanatory variables. Estimates for the secondary endpoints were reported as least squares means, differences in least squares means, 95% confidence intervals, and *p*-values for the treatment effect. At week 24, no secondary endpoint variables showed a statistically significant change at the 5% level.

In secondary exploratory analyses involving longitudinal measurements through week 52, mixed-effects models were also employed to estimate potential carryover effects. These included eGFR, blood pressure, and HbA1c, amongst others. Mean baseline (± SD) eGFR was 88.7 (± 48.5) ml/min, mean HbA1c was 61.7 (± 29.2) mmol/mol, and mean systolic blood pressure (SBP) was 132.3 (± 17.7) mmHg. None of the secondary endpoints reached statistical significance in these analyses (*p*-value > 0.1 for all) (**Supplementary Table 3**).

Baseline data for participants at each stage of the crossover phases of the trial are shown (**Table 1**). No significant baseline differences were observed between phases.

With regard to safety analysis, 37 adverse events (AEs) were observed during the study. Of these, 33/37 (89.2%) were nonserious adverse events, whilst four of 37 (10.8%) were serious adverse events (SAEs). The 37 AEs were experienced by 16 participants: 10 participants experienced 15 events in the placebo arm, and six participants experienced 22 events in the ursodeoxycholic acid arm. Three participants in the ursodeoxycholic acid arm experienced four SAEs (viral lower respiratory tract infection, diabetic ketoacidosis, alcohol dependence syndrome, and bowel resection). One participant experienced two SAEs (alcohol dependence syndrome and diabetic ketoacidosis) (see **Table 2**). The relatedness of the AEs and SAEs to study treatment is shown in **Table 2**. Discontinuation of study medication was reported by seven participants, and none withdrew from the study due to an AE or SAE. Liver function tests were performed at baseline and throughout the study as part of protocol-specified laboratory safety assessments at weeks 4, 24, 28, 32, and 52, with dedicated liver profile safety visits at weeks 4 and 32 and subsequent telephone safety follow-up. Gastrointestinal tolerability was not systematically monitored; however, all participants were informed of common known AEs associated with the medication, as indicated by the summary of product characteristics.

**Table 2:** Summary of treatment-emergent adverse events during the trial.

Outcome	Placebo (*n* = 15 events)	Ursodeoxycholic acid (*n* = 22 events)	Total (*n* = 37 events)
Overall safety			
Total adverse events	15	22	37
Nonserious adverse events	15 (100.0%)	18 (81.8%)	33 (89.2%)
Serious adverse events	0 (0.0%)	4 (18.2%)	4 (10.8%)
Adverse events leading to treatment discontinuation	0	0	0
Organ system			
Respiratory	3 (20.0%)	4 (18.2%)	7 (18.9%)
Gastrointestinal	1 (6.7%)	5 (22.7%)	6 (16.2%)
Genitourinary/renal	0 (0.0%)	5 (22.7%)	5 (13.5%)
Musculoskeletal/connective tissue	6 (40.0%)	4 (18.2%)	10 (27.0%)
Eye/ear/nose/throat	1 (6.7%)	0 (0.0%)	1 (2.7%)
Psychological	0 (0.0%)	1 (4.5%)	1 (2.7%)
Skin/subcutaneous tissue	0 (0.0%)	1 (4.5%)	1 (2.7%)
Endocrine	0 (0.0%)	1 (4.5%)	1 (2.7%)
Other	4 (26.7%)	1 (4.5%)	5 (13.5%)
Severity			
Mild	3 (20.0%)	5 (22.7%)	8 (21.6%)
Moderate	9 (60.0%)	13 (59.1%)	22 (59.5%)
Severe	2 (13.3%)	3 (13.6%)	5 (13.5%)
Not recorded/unknown	1 (6.7%)	1 (4.5%)	2 (5.4%)
Relationship to study drug			
Definite	5 (33.3%)	5 (22.7%)	10 (27.0%)
Probable	2 (13.3%)	0 (0.0%)	2 (5.4%)
Remote	0 (0.0%)	3 (13.6%)	3 (8.1%)
None/not related	8 (53.3%)	11 (50.0%)	19 (51.4%)
Not recorded/unknown	0 (0.0%)	3 (13.6%)	3 (8.1%)
Outcome			
Recovered	12 (80.0%)	19 (86.4%)	31 (83.8%)
Recovered with sequelae	0 (0.0%)	2 (9.1%)	2 (5.4%)
Continuing at end of study	3 (20.0%)	1 (4.5%)	4 (10.8%)

For severity, intensity was recorded for 35 of 37 events, with two events (one during placebo and one during ursodeoxycholic acid treatment) having no recorded intensity. For the relationship with study treatment, 19 events were recorded as having no relationship (“none”), whilst three events during UDCA treatment had no relationship assessment recorded.

## Discussion

In this first-ever clinical trial investigating the potential reno-protective effect of ursodeoxycholic acid added to standard of care in people with T1D and DKD who had persistent albuminuria despite RAS inhibition, we did not observe a significant reduction in UAER compared with placebo.

Our study has several strengths and limitations. A strength is the use of UAER, which is the gold standard measurement of urinary albumin excretion, as albuminuria has high short‐term within‐person variability (19). Another strength is the crossover design, which enables comparison of treatment effects within subjects, since each subject serves as their own control. This approach reduces interindividual variability between treatment groups, minimises the influence of covariates, and mitigates the allocation imbalance often seen with parallel designs (20). In addition, the risk of selection, performance, and observer bias was minimised through randomisation and blinding. We also included people on RAS inhibition with albuminuria, a group with significant residual cardiorenal risk. Indeed, there is a limited number of approved treatment options for this group of people to reduce DKD progression.

An important limitation is the relatively small number of participants (*n* = 31). However, this was in the context of a proof-of-concept study investigating the role of ursodeoxycholic acid as a potential treatment for DKD. The lack of participants with a higher degree of UAER may have potentially limited both the effect size and statistical power of our study. The median baseline UAER in our cohort was 30.4 μg/min, which lies at the lower end of the microalbuminuric range (20–200 μg/min). Although higher UAER could have potentially increased statistical power due to a greater effect size, even lower levels of albuminuria have been associated with DKD progression and CVD risk, which justifies treatment to reduce UAER and investigation of renoprotective measures in such cohorts (21–23). It is also worth noting that recent trials that demonstrated the impact of sodium–glucose cotransporter-2 inhibitors on albuminuria included subjects with lower levels of albuminuria (baseline ACR, < 4.5 mg/mmol) (24, 25) than those included in studies conducted in the past (26, 27). Indeed, the impact of albuminuria on cardiorenal risk emerges at thresholds within the normal range, with an inflection in risk at UACR > 10 mg/g (28–30).

Adherence to medications was assessed by pill counting and reinforced at each visit. We acknowledge that more specific indices of adherence, including direct measurement of ursodeoxycholic acid levels in serum, may be better measures of adherence. Good adherence in terms of ursodeoxycholic acid use in liver disease is often defined as > 80% use, which is associated with improvements in abnormal liver function tests. In our study, adherence was > 90% in people with DKD (31).

In type 1 diabetes, DKD progression risk persists despite current approved treatments, which are primarily based on RAS inhibition. Even with recent promising results with finerenone demonstrating benefits in reducing albuminuria, residual renal risk (as evidenced by residual albuminuria or renal function loss) can often persist in people with DKD and type 1 diabetes. There is therefore an urgent need for additional treatments to reduce renal risk in T1D (7).

Ursodeoxycholic acid at the dose we studied has been shown to improve endothelial function in people with CVD and heart failure independent of effects on blood pressure and lipids, has good systemic bioavailability, and is well tolerated with no serious adverse effects or participant withdrawals (15, 32). Indeed, endothelial dysfunction is implicated in the pathogenesis of albuminuria in people with diabetes (15). The effects of ursodeoxycholic acid on endothelial function in people with cardiovascular disease have been evaluated in a double-blind, placebo-controlled crossover study of 17 participants with stable chronic heart failure (stage II/III). Ursodeoxycholic acid treatment at 500 mg BID for 4 weeks improved endothelial function (14). In another trial including 11 participants with ischaemic heart disease, 6 weeks of ursodeoxycholic acid (dose, 13–19 mg/kg/day; ~ 1,000 mg/day) demonstrated beneficial effects on the endothelium (17). In both trials, participants were on RAS inhibitors and statins. Ursodeoxycholic acid was well tolerated, had good systemic bioavailability, and improved endothelial function independent of blood pressure or lipid levels.

We observed a numerical imbalance in total AEs, gastrointestinal AEs, and genitourinary/renal AEs between treatment phases, with a higher number of events reported with ursodeoxycholic acid. In terms of the possible relatedness of these AEs and SAEs to the study medication, this was considered plausible in 12 cases. Further longer-term studies are therefore required to ascertain the long-term safety and tolerability of ursodeoxycholic acid in people with diabetes and DKD. This may have also contributed to the attrition rate of seven participants who dropped out postrandomisation during the course of the study, and this dropout rate (~ 22%) could have also negatively impacted the power of the study to detect a significant difference in UAER despite our modified ITT approach.

In conclusion, in this proof-of-concept, single-centre, double-blind, placebo-controlled crossover trial, treatment with ursodeoxycholic acid over 24 weeks did not produce significant changes in UAER, eGFR, aortic pulse wave velocity (Ao-PWV), SBP, or HbA1c from baseline compared with placebo. The neutral result in this study with ursodeoxycholic acid in DKD may be due to a true absence of an effect or limitations in the statistical power of our study. Even though no significant impact was demonstrated, the biological effects of ursodeoxycholic acid make it a potentially attractive investigational agent for cardiovascular and renal pathology, especially when there is residual cardiorenal risk despite current standard of care. Further larger-scale research is warranted to explore possible clinical benefits and safety of ursodeoxycholic acid in people with DKD.

## Data Availability

The datasets presented in this study can be found in online repositories. The names of the repository/repositories and accession number(s) can be found below: The output generated from this study is available at the European Union Clinical Trials Register. EudraCT/CTIS number: 2015-003609-41.
