# Supplementary material for: The effects of ursodeoxycholic acid on albuminuria in people with type 1 diabetes and diabetic kidney disease: a proof-of-concept study
Source: Front Endocrinol (Lausanne). 2026 Sep 24;17:1908356. doi: 10.3389/fendo.2026.1908356 (PMC13650430; doi:10.3389/fendo.2026.1908356)
Supplement: Supplementary file 1 [file Table1.docx]

**Supplementary** **Table 1.** Baseline characteristics of all participants who were eligible for modified intention to treat analysis

| **Characteristic** | **Overall cohort (n=31)** |
| --- | --- |
| Age, years, median (IQR) | 54.2 (22.7–75.3) |
| Male sex, n (%) | 21 (67.7) |
| Ethnicity, n (%) |  |
| White | 27 (87.1) |
| Asian | 2 (6.5) |
| Black | 1 (3.2) |
| Mixed | 1 (3.2) |
| Diabetes duration, years, median (IQR) | 31.3 (11.8–59.5) |
| BMI, kg/m², mean ± SD | 26.9 ± 5.3 |
| UAER, μg/min, median (IQR) | 30.4 (15.3-61.3) |
| eGFR, mL/min/1.73 m², mean ± SD | 88.7 ± 48.5 |
| Systolic BP, mmHg, mean ± SD | 132.3 ± 17.7 |
| Diastolic BP, mmHg, mean ± SD | 76.8 ± 9.4 |
| HbA1c, mmol/mol (%, mean ± SD) | 61.7 ± 29.2 (7.8 ± 2.7) |
| Aortic pulse wave velocity, m/s, median (IQR) | 8.1 (6.6–9.8) |
| Total cholesterol, mmol/L, mean ± SD | 4.2 ± 0.9 |
| Triglycerides, mmol/L, median (IQR) | 1.0 (0.8–1.7) |

Data are presented as mean ± SD, median (IQR), or n (%), as indicated.
*Abbreviations: IQR* *Inter Quartile Range SD- standard deviation UAER = Urine Albumin Excretion Rate, eGFR = Estimated Glomerular Filtration Rate, SBP = Systolic blood pressure, HbA1c= Glycated Haemoglobin A1c*

**Supplementary Table 2.** The effect of Ursodeoxycholic acid on change from baseline in the primary endpoint and selected secondary endpoints

|  | **Estimate** | **Standard error** | **p-value** |
| --- | --- | --- | --- |
| Log UAER | -0.3 | 0.3 | 0.4 (-0.8, 0.3) |
| eGFR (ml/min/1.73m^2^) | 2.4 | 4.3 | 0.6 (-6.4, 11.2) |
| Ao-PWV (m/s) | 0.7 | 0.7 | 0.3 (-0.8, 2.1) |
| SBP (mmHg) | 5.7 | 3.9 | 0.2 (-2.2, 13.5) |
| HbaA1c (mmol/mol) | 8.0 | 9.7 | 0.4 (-11.7, 27.8) |

*Abbreviations: UAER = Urine Albumin Excretion Rate, eGFR = Estimated Glomerular Filtration Rate, SBP = Systolic blood pressure, HbA1c= Glycated Haemoglobin A1c, Ao-PWV = Aortic pulse wave velocity*

**Supplementary Table 3.** Mixed effects model to check for carryover and period effect on the primary endpoint and selected secondary endpoints

| **Log UAER** | **Estimate** | **Standard error** | **p-value (95% CI)** |
| --- | --- | --- | --- |
| Treatment | -0.1 | 0.1 | 0.5 (-0.3, 0.2) |
| Period | 0.1 | 0.1 | 0.3 (-0.1, 0.4) |
| Sequence | -0.5 | 0.6 | 0.4 (-1.7, 0.7) |
|  |  |  |  |
| **eGFR (ml/min/1.73m^2^)** |  |  |  |
| Treatment | 2.3 | 2.3 | 0.3 (-2.3, 6.9) |
| Period | -3.8 | 2.3 | 0.1 (-8.4, 0.8) |
| Sequence | -15.0 | 15.9 | 0.4 (-46.3, 16.3) |
|  |  |  |  |
| **Ao-PWV (m/s)** |  |  |  |
| Treatment | 0.4 | 0.4 | 0.3 (-0.4, 1.3) |
| Period | 1.1 | 0.4 | 0.01 (0.2, 1.9) |
| Sequence | -0.8 | 1.4 | 0.6 (-3.5, 1.9) |
|  |  |  |  |
| **SBP (mmHg)** |  |  |  |
| Treatment | -3.2 | 2.7 | 0.2 (-8.5, 1.9) |
| Period | -2.4 | 2.7 | 0.4 (-7.6, 2.8) |
| Sequence | 2.3 | 5.6 | 0.7 (-8.8, 13.3) |
|  |  |  |  |
| **HbA1c (mmol/mol)** |  |  |  |
| Treatment | 1.1 | 5.3 | 0.8 (-9.4, 11.5) |
| Period | 8.5 | 5.3 | 0.1 (-1.9, 18.9) |
| Sequence | -10.7 | 6.7 | 0.1 (-23.9, 2.6) |

*Abbreviations: UAER =Urine Albumin Excretion Rate, eGFR = Estimated Glomerular Filtration Rate, SBP = Systolic blood pressure, HbA1c= Glycated Haemoglobin A1c, Ao-PWV = Aortic pulse wave velocity*
